# Dietary Diversity among Preschoolers: A Cross-Sectional Study in Poor, Rural, and Ethnic Minority Areas of Central South China

**DOI:** 10.3390/nu11030558

**Published:** 2019-03-06

**Authors:** Jieying Bi, Chengfang Liu, Shaoping Li, Zhenya He, Kevin Chen, Renfu Luo, Zimeiyi Wang, Yanying Yu, Haiquan Xu

**Affiliations:** 1 Agricultural Information Institute of Chinese Academy of Agricultural Sciences, Beijing 100081, China; bijieying@caas.cn; 2 China Center for Agricultural Policy, School of Advanced Agricultural Sciences, Peking University, Beijing 100871, China; cfliu.ccap@pku.edu.cn (C.L.); zhe6@masonlive.gmu.edu (Z.H.); luorf.ccap@pku.edu.cn (R.L.); 3 China Academy for Rural Development, Zhejiang University, Hangzhou 310058, China; Kzchen@zju.edu.cn (K.C.); 11722013@zju.edu.cn (Y.Y.); 4 International Food Policy Research Institute, East and Central Asia Office, Beijing 100081, China; Z.Wang@cgiar.org; 5 School of Economics and Management, Jiangxi Agricultural University, Nanchang 330045, China; 6 Institute of Food and Nutrition Development, Ministry of Agriculture and Rural Affairs, Beijing 100081, China; xuhaiquan@caas.cn

**Keywords:** dietary diversity, preschoolers, ethnic minorities, rural China

## Abstract

The aim of this study was to document the dietary diversity status of preschool children in poor, rural, and ethnic minority areas of Central South China and examine its associated factors both at home and in preschools. A cross-sectional study including 1328 preschool children aged three or five years from two nationally designated poverty counties in Hunan Province was conducted. A dietary diversity score (DDS) was constructed to measure the dietary patterns based on the 24 h recall method. The mean DDS among the sample children was 5.77 (95% confidence interval: 5.70–5.83, range 1 to 9) with a standard deviation of 1.22. Both household characteristics (including the education level of the child’s primary caregiver and the nutritional knowledge of the caregiver) and preschool factors (including the nutritional knowledge of the child’s preschool principal and teachers, nutritional training to children, and the preschool kitchen manager) were positively associated with children’s DDS. The dietary diversity status of children in poor, rural, and ethnic minority areas of Central South China is much lower than that of their peers in other areas. Nutritional education should be provided to caregivers, preschool staff, and children to narrow the gap.

## 1. Introduction

The Sustainable Development Goals (SDGs) call for efforts to eliminate world hunger and malnutrition in all forms by 2030, and ensure the access of all people, especially children, to nutritious and abundant food. The focus on children under five years old has become an internationally standard practice when examining progress toward meeting the SDG targets or indicators [1]. Statistics show that, by 2017, millions of children under the age of five were still suffering from various forms of malnutrition. The numbers of children suffering from stunting, wasting, and being overweight were 150 million, 50 million, and 38 million, respectively [2]. The majority of these children were concentrated in poor, rural areas of developing countries [3,4]. We cannot afford to leave these children behind as malnutrition has various adverse effects, such as suboptimal brain development [5], immune deficiency [6], and a high risk of morbidity and mortality [7]. According to Black et al., malnutrition is estimated to contribute to more than 3.1 million child deaths annually, or 45% of all child deaths [8].

Why are so many children malnourished? There are many reasons behind child malnutrition, one of which is a lack of dietary diversity [9,10]. A large and growing body of literature has documented that a varied diet is significantly associated with micronutrient adequacy and leads to positive health outcomes [11,12,13,14,15,16,17]. By contrast, a less diversified diet may increase the risks of being stunted and underweight [18,19], and even cause cognitive deficits [20]. Therefore, to design interventions to effectively reduce child malnutrition by providing a diversified diet, it is necessary to understand the status of dietary diversity among children and its associated factors.

China is a good place to study dietary diversity in children. Although nutrition in China has considerably improved since the 1970s, a large group of children still suffer from malnutrition. This is especially true in poor, rural, and ethnic minority areas in China. The prevalence of malnutrition was 19.2% among children under five years in poor areas of China in 2016 [21]. Over 65% of children aged 12–24 months suffered from vitamin D deficiency in 2003 [22].

Two studies explored the dietary diversity status of Chinese children and examined its correlated factors, such as household sociodemographic characteristics, children’s eating habits, and micronutrient inadequacy [23,24]. To the best of our knowledge, however, none of these studies were undertaken in the poor, rural areas of China, where children suffer more, if not the most, from malnutrition. In addition, these rural regions have a high concentration of ethnic minorities. Another concern is the small sample size. For example, Jiang et al. included only 697 children aged three to seven years old, so their study may be subject to the problem of a small sample size [23]. The correlation between dietary diversity and age was found in many settings [25,26]. Although Meng et al. sampled 2012 children, the sample children ranged in age from 3 to 17 years [24]. This means that there was only a small number of children in each age group, complicating the examination of the heterogeneity of dietary diversity across age groups, especially the status of preschool-aged children. Due to data limitations, most existing studies on children’s dietary diversity focused on the role of household factors; few studies have analyzed the impact of school factors. In short, in this context in China, the dietary diversity status of preschool children in poor, rural areas is still unclear, as is the role that preschools might play in children’s dietary diversity.

In this paper, we contribute to the existing literature by filling the gaps mentioned above. To do thus, we first documented the dietary diversity status of preschool children in poor, rural, and ethnic minority areas of Central South China. Then, we examined the correlation between the dietary diversity of preschool children and its associated factors from both the household side and the preschool side.

## 2. Subject and Approach

### 2.1. Sample Selection

We collected the data in September 2018 in two nationally designated poverty counties in the Xiangxi Autonomous Prefecture, Hunan Province in the south central part of China. According to national statistics, the rural per capita disposable income in Xiangxi was 8273 yuan in 2017, which is similar to China’s rural per capita disposable income in 2012 [27,28]. The data used for this study were obtained from the baseline survey of a preschool nutrition pilot program launched by the Xiangxi prefecture government, with support from the World Food Program (WFP). Because the baseline survey was conducted prior to any intervention associated with the pilot program, the intervention can be ignored here.

The sample includes 26 preschools from 15 townships across the two project counties. Of these, 10 preschools were from county L, whereas the remaining 16 were from county Y. Within each sample preschool, all children aged three or five years who attended the preschool on the survey day were included in the sample. In total, we surveyed 1334 preschoolers. Among them, six children did not provide information on food consumption, thus the full sample size of this study was 1328.

### 2.2. Data Collection

The survey team collected three types of information: Dietary data of sample preschoolers (as measured by detailed food consumption within the past 24 h at home and in preschool), sociodemographic survey for children and caregivers (including socioeconomic and demographic characteristics), and preschool data (as measured by class characteristics and preschool staff characteristics).

#### 2.2.1. Dietary Data

Children’s dietary data were obtained by trained enumerators using two questionnaires. Both questionnaires used the 24 h recall method. In this study, one questionnaire aimed to ask the primary caregivers (mostly grandparents or parents) what the children ate at home as well as food eaten at restaurants or other shops over the past 24 h. The other questionnaire aimed to ask the preschool kitchen managers what the children ate at preschools over the past 24 h. As such, we collected detailed food consumption of each child both at home and at preschool over the past 24 h, which allowed us to measure the children’s total dietary consumption within the past 24 h.

Dietary diversity score (DDS) and food variety score (FVS) are the most commonly used tools for measuring dietary diversity. However, studies showed that DDS is more effective in terms of casting nutritional adequacy than FVS [29,30]. Therefore, following the Guidelines for Measuring Household and Individual Dietary Diversity provided by the Food and Agriculture Organization of the United Nations [31], we used dietary diversity score (DDS) to measure the dietary diversity of children within the past 24 h, based on the dietary data collected from primary caregivers and preschool kitchen managers. DDS was based on nine diverse food groups. Table 1 shows the detailed food group classification and example food items in each group. The DDS was calculated by counting the number of food groups that a child consumed in the past 24 h without consideration of a minimum quantity requirement for any food group. Any individual food item in each food group consumed by a child earns one point for their dietary diversity score, but different individual food items consumed in the same group are not be counted repeatedly. Therefore, DDS ranges from 0 to 9.

#### 2.2.2. Sociodemographic Survey

The sociodemographic survey was administered to the primary caregivers by trained enumerators to collect basic demographic information on the child, the primary caregiver, the parents, and other household members. The survey also asked a series of basic questions about the migration status of the children’s parents as well as the household conditions. To determine the social economic status of the households, the possession status of a list of 13 durable assets/goods was used. To measure the nutritional knowledge of caregivers, the survey asked them a set of 10 multiple choice questions about macronutrients and micronutrients (e.g., protein, calcium, iron) as well as sources and functions of nutrients. One point was assigned for each correct answer for a total possible score of 10 points.

#### 2.2.3. Preschool Data

Information about preschool factors was gathered during interviews with preschool staff. The teachers were asked whether they taught children any nutritional knowledge in class over the past 12 months. Similarly, enumerators asked the principals whether the preschool held any nutrition-related activities for children’s primary caregivers in the past 12 months. The survey collected information about the number of students and teachers in each class, preschool staff education levels, and whether the staff attended any child nutrition forum/training in the past 12 months. We asked the preschool principal and teacher the same set of 10 multiple choice questions to measure their nutritional knowledge, just as we did with the primary caregivers.

### 2.3. Statistical Analysis

The database was established with Excel version 2016 (Microsoft Corporation, Redmond, WA, USA) and analysis was conducted with Stata version 15.1 (Stata Corporation, College Station, TX, USA). The household asset index was computed using the principal component analysis. *p* < 0.05 was considered statistically significant. The statistical significance of differences of all outcomes by subgroup populations was assessed using student’s *t*-test in Stata (Stata Corporation, College Station, TX, USA). Descriptive analysis was performed for continuous variables, and the results are shown as the mean and 95% confidence interval (CI). The predictors of DDS were explored by linear regression.

### 2.4. Ethics Statement

This study received ethical approval from the International Food Policy Research Institute Institutional Review Board (IRB) (DSG-18-0837). All participating children provided their consent for their involvement in the study. All legal guardians of children and school staff involved in the study provided written consent before their participation.

## 3. Results

### 3.1. DDS and Associated Household Factors

Table 2 provides the characteristics and sociodemographic information of the study population. A total of 1328 children (88.5% ethnic minorities) aged three or five years were included in the study. For the sample children, the mean DDS was 5.77 (95% CI: 5.70–5.83) with a SD of 1.22. The majority of fathers, mothers, and caregivers of children had an education level of junior high school or below. The proportion of children who are left-behind was high in the study, as 941 out of 1328 children (or 71%) had at least one parent not at home. Children whose caregivers had a lower education level and insufficient nutritional knowledge, and whose household owned fewer assets, had significantly lower DDS.

### 3.2. DDS and Associated Preschool Factors

Table 3 shows where the study population had breakfast, lunch, and supper. Although only 0.61% of preschoolers had supper in preschool, the proportions of children that had breakfast and lunch in preschool are 61.32% and 98.77%, respectively. This implies an important role played by preschool in child dietary diversity.

When examining the characteristics of preschools, we found that children who enrolled in public preschools and/or who received nutritional education in preschools had a significantly higher DDS (Table 4). Children in preschools where the principals and teachers had poor nutritional knowledge had a significantly lower DDS. Preschool kitchen managers who were well-educated and had received nutritional training in the past 12 months were more likely to contribute to a higher DDS among children.

### 3.3. Linear Regression Model of Predictors of Child DDS

To examine which factors from the household or the preschool are associated with children’s DDS, we conducted a multivariate analysis. Table 5 shows the results of the linear regression of predictors of DDS among the sample children. Both caregivers’ and preschool staff’s nutritional knowledge and education were positively associated with children’s DDS. Receiving nutritional education in preschool was also positively associated with child DDS.

### 3.4. Consumption of Each Food Group

**Figure 1:**
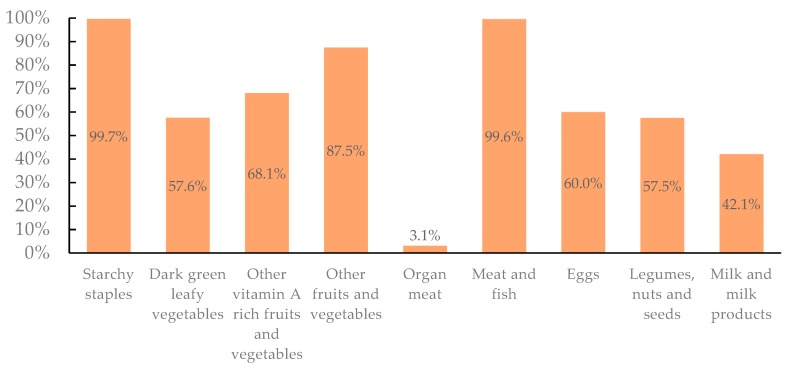
Percentage of children consuming each food group.

In this study, the most frequently consumed food groups were starchy staples (99.7%), followed by meat and fish (99.6%), other fruits and vegetables (87.5%), and other vitamin A-rich fruits and vegetables (68.1%). In comparison, the groups of dark green leafy vegetables (57.6%); legumes, nuts, and seeds (57.5%); and milk and milk products (42.1%) were less likely to be consumed (Figure 1).

## 4. Discussion

Several researchers documented the importance of dietary diversity in a healthy and balanced diet [11,17], and the consequence of an unvaried diet [32,33,34,35]. The reliability of dietary diversity as a proxy of child nutrient status and its potential as a means of measuring food security has been emphasized by earlier studies [12,36,37]. The simplicity, efficiency, and reasonable accuracy of the dietary diversity score have made DDS a preferred tool for measuring the nutrient inadequacy of an individual’s diet. In the present study, the DDS based on nine food groups was used to assess the dietary diversity among 1328 preschool children in poor, rural, and ethnic minority areas in China. The DDS mean in our study was 5.77, which is significantly lower than the dietary diversity scores reported in other similar studies among Chinese children—for example, 6.1 in Meng et al. [24], 6.8 in Zhao et al. [10], and 7.4 in Jiang et al. [23].

Variations in the results of the DDS across studies may arise from the different study samples and differences in the measurement of DDS. Zhao et al. and Jiang et al. conducted their studies among children mostly from urban areas [10,23], where children may have better access to a variety of foods than their peers in rural areas [38]. Living in urban areas also means a higher household income and consequently a higher proportion of household expenditure allocated to food [39]. These factors provide potential reasons for the higher DDS in their studies. In Meng et al. [24], the sample children were aged 3–17 years. Although 64% of their sample children were from rural areas, they used a three-day recall to calculate DDS. Given that using a 24 h recall period does not always accurately reflect an individual’s dietary habits, a longer recall might be better. This might explain why a higher DDS was observed in Meng et al. [24]. However, a longer recall period may also lead to a higher recall bias [40]. With the large sample size in our study, 24 h recall can accurately reflect individual diet [41]. Li et al. reported a DDS of 4.18 among Chinese children aged 2 to 17 years [34]. The study employed a quantity threshold of at least 25 g for each food sub-category when measuring DDS, which might be the main reason for the low DDS in their study. However, the methodology (e.g., food groups based on 13 sub-categories; different points allocated to different sub-categories of food) used to assess dietary diversity by Li et al. differed substantially from others, which complicates its comparison with other studies. To date, a lack of international consensus on the methodology of measuring dietary diversity, including food group classification, minimum quantity requirement for food consumption, and reference time, makes comparison between studies problematic.

The associations between household-level factors and children’s dietary diversity scores were examined in the study. Measuring socioeconomic status in low-income settings can be complicated and sometimes imprecise due to monthly fluctuations in income and reporting bias [42]. The number of durable assets owned by households was thus used as a proxy for household socioeconomic status (SES) and its link with DDS has been demonstrated in some research [11,43]. Children whose families own more household assets tend to receive a higher dietary diversity score—a result similar to the positive association found between DDS and SES background in other studies [19,42]. No significant association was found between DDS and sex in our study, consistent with a previous study among preschoolers [23]. This lack of a sex gap implies there is no difference in the intra-household food allocation between boys and girls. No strong association was found between the ethnicity of children (Han vs. non-Han) and their DDS. This suggests that ethnic minority children do not have a less diversified diet than their Han peers. We also found that DDS was almost the same across the two age groups (three years old and five years old). Some studies showed that DDS is negatively associated with age, which may reflect poorer appetite among older subjects [42,44]. Neither maternal education nor paternal education had any significant association with children’s DDS, which is inconsistent with many previous studies [45,46]. However, the education of caregivers, most of whom were children’s grandparents, was significantly associated with the dietary diversity of children in the study. This might have something to do with the fact that about 71% of children in our research had at least one parent working outside the home as migrants. When examining the nutritional knowledge of the caregivers, our data show that caregivers with better nutritional knowledge have children with significantly more diversified diets.

In addition to the household-level factors, we also explored the association of preschool factors with children’s dietary diversity as most of preschoolers in our study had breakfast or lunch in the preschool. Our data showed that children attending publicly owned preschools have a significantly higher DDS than children in private schools. An underlying reason for this might be that private preschools tend to offer a less diversified diet to children due to cost considerations. This is more likely to be the case in rural areas, where income is very low. However, the number of students (as a proxy of scale) and student‒teacher ratio in preschools were not associated with DDS in our study. There is no economy of scale in the study area when it comes to children’s dietary diversity in preschools. Similar to the results of caregivers’ nutritional knowledge, the nutritional knowledge of both preschool principals and teachers was positively associated with preschoolers’ dietary diversity. Preschoolers whose principals have higher than average nutritional knowledge had a mean DDS of 5.88 compared to those whose principals have lower than average nutritional knowledge with a mean DDS of 5.68. Principals’ role in the food procurement process may explain the difference in children’s DDS, as principals with better nutritional knowledge are more likely to purchase foods that are rich in micronutrients. Preschoolers whose teachers have higher than average nutritional knowledge had a mean DDS of 5.85 compared to those whose teachers have lower than average nutritional knowledge with a mean DDS of 5.70.

Some studies have highlighted the importance of nutritional education in improving the dietary diversity of students [47,48]. This study showed that there was no association between whether the preschool provided nutritional education to caregivers and the children’s DDS. One possible reason is that the intensity of nutritional education might be too low to alter caregiver behaviors. Only five preschools held a nutritional education activity for primary caregivers in the year prior to the survey. However, a significant association was found between preschools providing nutritional education to the students and the children’s DDS. This result is consistent with other studies showing that the dietary quality and diversity of students significantly improved with health and nutritional education in school [47,49].

Regarding the variation in the consumption of different food groups, many reasons have been proposed to explain why people consume certain food groups over the others [50]. Two important reasons might be costs and accessibility [38,50]. In the study area, a majority of farm households (where our sample children are from) are engaged in planting staple food crops and vegetables for self-consumption. Even if they do not produce them by themselves, they could easily get them from the local markets at affordable prices [51]. Similarly, although meat, fish, and eggs are relatively expensive, they are also readily accessible from the local markets. And there has been a long-held perception that these food groups are good sources of nutrients for children. Therefore, caregivers tend to feed children with these food groups whenever possible. By contrast, although more and more people have realized that milk and milk products are also good sources of nutrients, they are relatively expensive and some people are not used to taking them. In fact, low consumption (15%) of milk has been observed in rural China in 2011 [52]. As far as animal organs are concerned, although they are used for food purpose in China, many parents are still concerned that organ meat is not safe or healthy enough for children [53]. This may explain why an extremely low portion of children consumed organ meat in our study.

To the best of our knowledge, the present study is the first to exclusively assess the dietary diversity status of preschoolers in poor, rural, and ethnic minority areas in China. Most of the existing studies on child dietary diversity focused on urban areas, which means the nutritional condition of poor, rural, remote areas is not as well understood. Our study fills that gap by providing a comprehensive assessment of the dietary diversity of preschool children in remote, poor, rural, and ethnic minority areas. In addition to household factors, we also examined the association of preschool factors with children’s dietary diversity—a particularly new area of study that has been less explored in the literature.

There are also some limitations in this study that should be considered. First, this was only a cross-sectional study, thus causal relationships between children dietary diversity and its correlated factors cannot be determined. Second, the sample population in this present study is limited to two counties of one province, making it difficult to generalize the findings to other regions within the country.

The dietary diversity status of preschool children in poor, rural, and ethnic minority areas in China is very low, much lower than their peers in other areas. To achieve the SDGs in China, efforts should be made to increase the dietary diversity of preschool children in these areas to improve their nutritional status. When designing interventions with the goal of improving child dietary diversity in these areas, both household and school factors should be considered. The effects of interventions that provide nutritional education to caregivers, preschool staff, and children themselves should be examined in future prospective studies.

## Figures and Tables

**Table 1:** Nine food groups of dietary diversity scores.

Food Group	Examples ^1^
1. Starchy staples	Cereals (corn/maize, rice, wheat, sorghum, millet or any other grains or foods made from these (e.g., bread, noodles, porridge, or other grain products) and white tubers and roots (e.g., white potatoes, white yam, or other foods made from roots)
2. Dark green leafy vegetables	Dark green/leafy vegetables, such as, Chinese cabbage, spinach, coriander, rape, etc.
3. Other vitamin A rich fruits and vegetables	Pumpkin, carrot, squash, or sweet potato that are orange inside + other locally available vitamin A rich vegetables (e.g., red sweet pepper), cantaloupe, apricot (fresh or dried), dried peach, and 100% fruit juice made from these
4. Other fruits and vegetables	Other vegetables (e.g., tomato, onion, eggplant) + other locally available vegetables, such as cabbage, green pepper, lettuce, radish, garlic, tomato, and other fruits, including wild fruits and 100% fruit juice made from these
5. Organ meat	Liver, kidney, heart, or other organ meats or blood-based foods
6. Meat and fish	Beef, pork, lamb, goat, rabbit, game, chicken, duck, other birds, insects, fresh or dried fish or shellfish
7. Eggs	Eggs from chicken, duck, or any other egg
8. Legumes, nuts and seeds	Cowpea, peanut, dried beans, dried peas, lentils, seeds or foods made from these
9. Milk and milk products	Milk, yogurt or other milk products

^1^ Examples are adapted from the Guidelines for Measuring Household and Individual Dietary Diversity [31].

**Table 2:** The dietary diversity score (DDS) and preschooler’s socio-demographic characteristics.

Socio-Demographic Characteristics		*N*	DDS	95% CI	*p*
Gender	Female	643	5.79	(5.79, 5.89)	0.454
	Male	685	5.74	(5.63, 5.83)	
Age (years)	3-year old	537	5.78	(5.67, 5.88)	0.896
	5-year old	629	5.77	(5.68, 5.87)	
Ethnicity	Non-Han	1180	5.75	(5.68, 5.82)	0.063
	Han	147	5.95	(5.74, 6.15)	
Father’s education	Junior high school or below	1078	5.77	(5.70, 5.84)	0.410
	Senior high school o above	166	5.86	(5.70, 5.84)	
Mother’s education	Junior high school or below	1010	5.75	(5.67, 5.82)	0.093
	Senior high school o above	159	5.92	(5.73, 6.12)	
Caregiver’s education	Junior high school or below	1162	5.74	(5.67, 5.81)	0.029
	Senior high school o above	109	6.01	(5.77, 6.25)	
Caregiver’s nutritional knowledge	Above the mean	731	5.90	(5.81, 5.99)	<0.001
	Below the mean	597	5.60	(5.50, 5.70)	
Parental migration status	Both parents are at home	387	5.84	(5.71, 5.97)	0.180
	At least one parent is not at home	941	5.74	(5.66, 5.81)	
Household asset index	The lowest 1/3	443	5.65	(5.54, 5.76)	0.006
	The middle 1/3	444	5.75	(5.63, 5.86)	
	The highest 1/3	441	5.91	(5.79, 6.01)	

**Table 3:** Eating locations of preschoolers.

	Breakfast	Lunch	Supper
Home	38.68%	1.23%	99.39%
Preschool	61.32%	98.77%	0.61%

**Table 4:** The DDS and preschool characteristics.

Preschool Characteristics		*N*	DDS	95% CI	*p*
Preschool ownership	Public	279	6.02	(5.87, 6.18)	<0.001
	Private	1049	5.70	(5.63, 5.77)	
Number of students	Above the mean	869	5.72	(5.64, 5.80)	0.067
	Below the mean	459	5.85	(5.73, 5.96)	
Student-teacher ratio	Above the mean	671	5.80	(5.71, 5.90)	0.258
	Below the mean	657	5.73	(5.63, 5.82)	
Principal’s nutritional knowledge	Above the mean	575	5.88	(5.78, 5.98)	0.004
	Below the mean	753	5.68	(5.59, 5.77)	
Teacher’s nutritional knowledge	Above the mean	579	5.85	(5.75, 5.95)	0.024
	Below the mean	749	5.70	(5.61, 5.79)	
Preschool kitchen manager’s education	Junior high school or below	501	5.58	(5.47, 5.69)	0.035
	Senior high school or above	392	5.76	(5.63, 5.88)	
Nutrition training for preschool kitchen manager in the past 12 months	Yes	407	5.80	(5.67, 5.92)	0.041
	No	528	5.63	(5.53, 5.73)	
Giving nutritional education to caregivers	Yes	212	5.65	(5.48, 5.83)	0.129
	No	1116	5.79	(5.71, 5.86)	
Giving nutritional education to students	Yes	953	5.92	(5.84,6.00)	<0.001
	No	375	5.38	(5.27, 5.50)	

**Table 5:** Linear regression model of predictors of DDS.

Variables ^1^	*B*	95% CI	*p* ^2^
Caregiver’s education	0.20	(0.04, 0.36)	0.017
Caregiver’s nutritional knowledge	0.24	(0.07, 0.40)	0.005
Household asset index	0.05	(0.01, 0.09)	0.008
Preschool ownership	0.08	(0.02, 0.15)	0.012
Principal’s nutritional knowledge	0.48	(0.27, 0.69)	<0.001
Teacher’s nutritional knowledge	0.29	(0.10, 0.47)	0.002
Preschool kitchen manager’s education	0.16	(−0.06, 0.38)	0.166
Nutrition training for preschool kitchen manager in the past 12 months	−0.08	(−0.28, 0.12)	0.435
Giving nutritional education to students	0.53	(0.33, 0.74)	<0.001

^1^ The caregiver’s and kitchen manager’s education are defined by junior high school or below, and senior high school or above, and assigned values as 0 and 1, respectively. The caregiver’s nutritional knowledge, principal’s nutritional knowledge, and teacher’s nutritional knowledge were categorized as two groups: Those above the average take the value of 1, and 0 otherwise, respectively. Household asset index was a continuous variable. Preschool ownership was categorized for two groups with the public ownership taking a value of 1, and 0 otherwise. Nutrition training for preschool kitchen managers in the past 12 months and giving nutritional education to students were categorized for two groups as yes and no, and assigned values as 1 and 0, respectively. ^2^ *p* values were calculated based on standard errors clustering at the preschool level.
